# An AI methodology to reduce training intensity, error rates, and size of neural networks

**DOI:** 10.3389/fncom.2025.1628115

**Published:** 2025-10-21

**Authors:** Thaddeus J. A. Kobylarz

**Affiliations:** Retired, Murray Hill, NJ, United States

**Keywords:** non-linearly separable neurons, far less training, much smaller neural networks, far less power for training, no network hallucinations

## Abstract

Massive computing systems are required to train neural networks. The prodigious amount of consumed energy makes the creation of AI applications significant polluters. Despite the enormous training effort, neural network error rates limit its use for medical applications, because errors can lead to intolerable morbidity and mortality. Two reasons contribute to the excessive training requirements and high error rates; an iterative reinforcement process (tuning) that does not guarantee convergence and the deployment of neuron models only capable of realizing linearly separable switching functions. tuning procedures require tens of thousands of training iterations. In addition, linearly separable neuron models have severely limited capability; which leads to large neural nets. For seven inputs, the ratio of total possible switching functions to linearly separable switching functions is 41 octillion. Addressed here is the creation of neuron models for the application of disease diagnosis. Algorithms are described that perform direct neuron creation. This results in far fewer training steps than that of current AI systems. The design algorithms result in neurons that do not manufacture errors (hallucinations). The algorithms utilize a template to create neuron models that are capable of performing any type of switching function. The algorithms show that a neuron model capable of performing both linearly and nonlinearly separable switching functions is vastly superior to the neuron models currently being used. Included examples illustrate use of the template for determining disease diagnoses (outputs) from symptoms (inputs). The examples show convergence with a single training iteration.

## 1 Introduction

Current neural networks essentially employ the McCulloch–Pitts (McCulloch and Pitts, 1943) neuron model, introduced over 80 years ago. The original McCulloch-Pitts neuron model is defined by the following two equations:


(1) $$\begin{array}{l} \text{u}=\sum_{\text{i}=\text{1}}^{\text{n}}{\text{w}}_{\text{i}}{\text{x}}_{\text{i}} \end{array}$$

where: $\left({\text{w}}_{1},{\text{w}}_{2},\ldots ,{\text{w}}_{\text{n}}\right)=$ **w**″ is an analog vector of synaptic weights,

(x_1_, x_2_, …x_*n*_) = **x** is a binary vector of inputs,

u is an analog summation result.


(2) $$\begin{array}{l} \text{y}=\{\begin{array}{c} 0,\;\text{if }\text{u}<\theta \\ 1,\;\text{if }\text{u}>\theta \end{array} \end{array}$$

where: “y” is a binary output,

“u” is computed from Equation 1.

“θ” is a known threshold used to compute “y.”

In order to improve neural network performance, Equation 1 was extended with the following representation (Alzahrani and Parker, 2020).

**Figure 1:**
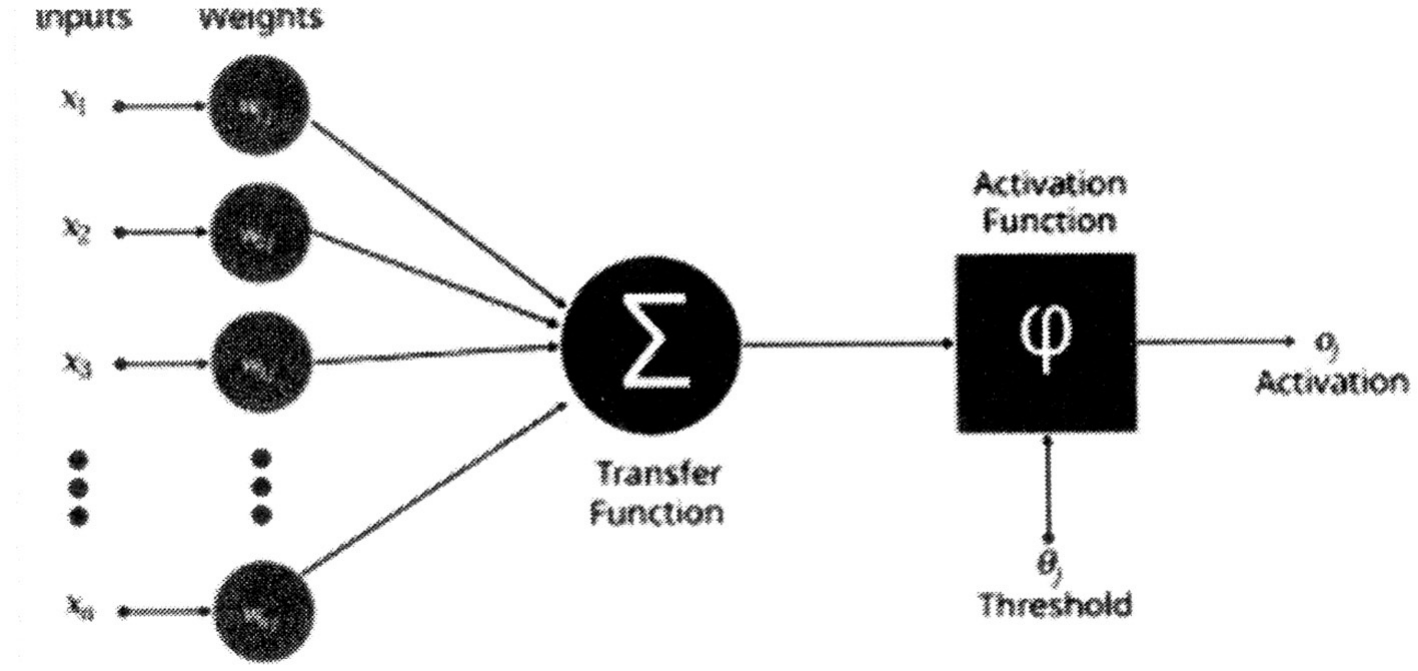
Artificial neuron structure (Alzahrani and Parker, 2020).

The corresponding equation (Alzahrani and Parker, 2020) for Figure 1 is:


(3) $$\begin{array}{l} {\text{a}}_{\text{y}}=\varphi \left(\sum_{\text{i}=\text{1}}^{\text{n}}{\text{w}}_{\text{i}}{\text{x}}_{\text{i}}\right) \end{array}$$

A variety of activation functions (**φ**) exist. If the activation function is a binary step function with a threshold comparison, Equation 3 represents the composite of Equations 1, 2. Instead of using a linear or stair-step function that results in binary classification (0 or 1), some neuron models use a sigmoid function (Tanaka, 2020) to assist in generating nonlinear separations. The function generates binary, digital outputs for continous inputs. Continuous inputs is inconsistent with accepted neurological neurons' properties. Neurological neurons' input signals are binary and they perform switching functions.

Observe that the transfer function (**σ**) is the same as in Equation 1. Hence, all realizations of switching functions remain limited to the linearly separable class for Equation 3. It is very significant that the neurological neuron can perform both linearly separable and nonlinearly separable switching functions (Kobylarz and Kobylarz, 2023). Another neurological neuron inconsistency exists in that the threshold of the above neuron models vary according to the switching function realization. The neurological neuron's threshold has a per unit value of “1” and is constant (Kobylarz and Kobylarz, 2023). If the above neuron model (Equation 3) possessed the neurological constant threshold, it would not be able to perform an “and” function. A neuron model, derived from the Kobylarz-Bradley template, does not have the previously cited inconsistencies with neurological neurons.

Another weakness of the current AI methodology is “forgetting” data. During training, data could be overwritten to cause the forgetting. In the proposed methodology, data is permanently stored; unless commanded to change by a user.

A template has been conceived to generate (complete) neuron models capable of performing all linearly separable and nonlinearly separable switching functions, It was first disclosed in 1967 (Kobylarz and Bradley, 1967) and therefore is ascribed the name of the 1967 paper's authors Kobylarz-Bradley neuron model template (Kobylarz and Bradley, 1967).


(4) $$\begin{array}{l} {\text{F}}_{\text{n}}(\{X\})=\underset{\text{linear part}}{\underset{︸}{\underset{1@\text{ a time}}{\underset{︸}{\sum_{\text{i}=1}^{\text{n}}{\text{W}}_{\text{i}}{\text{X}}_{\text{i}}+}}}}\underset{\text{nonlinear part}}{\underset{︸}{\underset{2\;@\text{ a time}}{\underset{︸}{\sum_{\text{i}=1}^{\text{n}-\text{1}}{\text{X}}_{\text{i}}(\sum_{\text{j}=\text{i}+\text{1}}^{\text{n}}{\text{W}}_{\text{ij}}{\text{X}}_{\text{j}})}}+\underset{3\;@\text{ a time}}{\underset{︸}{\sum_{\text{i=1}}^{\text{n}-2}{\text{X}}_{\text{i}}(\sum_{\text{j}=\text{i}+\text{1}}^{\text{n}-1}{\text{X}}_{\text{k}}(\sum_{\text{k}=\text{j}+\text{1}}^{\text{n}}{\text{W}}_{\text{ijk}}{\text{X}}_{\text{k}}))}}+\begin{array}{c} \;\\ \;\\ \ldots \\ \;\\ \ldots \end{array}+\underset{\text{n }@\text{ a time}}{\underset{︸}{\begin{array}{c} \;\\ {\text{W}}_{\text{1}\ldots \text{n}}{\text{X}}_{\text{1}}\ldots {\text{X}}_{\text{n}}\\ \; \end{array}}}}} \end{array}$$

where: F_n_ (**{X**}) is the neuron model's threshold function,

X_m_ is a variable that corresponds to the model's switching function input variable,

W_p_ is a weight assigned to a product of one or more X_m_,

**{X**} = {X_1_, …, X_n_} is the set of variables.

The template first identifies a summation of one variable at a time; the linear part. It next identifies a summation of products having two variables at a time. This continues until the product of all the variables of the set is included. The quantity of products for the summations that precede a plus (“+”) sign of Equation 4 is the combination of “n” variables taken “m” (m ≤ n) at a time:


(5) $$\begin{array}{l} {}[\begin{array}{c} \text{n}\\ \text{m} \end{array}]\text{=}\frac{\text{n!}}{\text{m!(n-m)!}} \end{array}$$

The product of a weight times its associated variables is a “term” of Equation 4. Having a weight (W_p_) equal to zero signifies that the term does not appear in a neuron model's threshold equation. For a complete explanation of the Kobylarz-Bradley neuron model template, including a proof that all switching functions can be represented by the template, see Kobylarz and Kobylarz (2023). This property has a far reaching significance. It implies that each switching function output for a neural network can be realized by a single neuron model.

It is important to understand that the neuron model Equation 4 is not a neuron model. Algorithms are presented to provide the means for extracting a neuron model from the template. These algorithms and examples are provide later.

Gidon et al. (2020) studied pyramidal neurons, which comprise approximately two-thirds of all neurons in the mammalian cerebral cortex, and therefore play the most important role in many cognitive and motor functions. They studied these most populous neurons from layers 2/3 of the human cerebral cortex *ex vivo* in an attempt to determine how the brain of the human species differs from those of other animals, e.g. rodents. Through the use of somatodendritic recordings they discovered previously unknown properties of the highly complex activity of these neurons. They described a class of calcium-mediated dendritic action potentials (dCaAPs) which occur in a graded, rather than all-or-none fashion, being sharply tuned to the amplitude of the stimulus. For threshold level stimuli the dCaAPs amplitudes are maximal, but become reduced with stronger stimuli. Such a relationship enables the human brain to have “linearly non-separable” (nonlinearly separable), rather than the conventionally adopted linearly separable multilayered network functionality.

This group utilized a compartmental model of L2/3 neurons to replicate dCaAP behavior in dendrites. A very important observation was that although each synaptic pathway could induce dCaAPs by itself, when 2 or more neurons fired simultaneously, the amplitude was reduced. These results suggest that there must be a balance between excitatory and inhibitory inputs to generate these action potentials; this supports the exclusive OR (XOR) logic operation for dCaAPs in the human cerebral cortex. The XOR function cannot be performed by a single linear neuron model (Minsky and Papert, 1972).

The emulation of the XOR function in a conventional neural sigmoid network has been reported (Data Science). The reported results were “I attempted to create a 2-layer network, using the logistic sigmoid function and backprop, to predict xor. My network has 2 neurons (and one bias) on the input layer, 2 neurons and 1 bias in the hidden layer, and 1 output neuron. To my surprise, this will not converge. if I add a new layer, so I have a 3-layer network with input (2 + 1), hidden1 (2 + 1), hidden2 (2 + 1), and output, it works. Also, if I keep a 2-layer network, but I increase the hidden layer size to 4 neurons + 1 bias, it also converges.” Observe that at least two layers and 4 neurons +1 bias are necessary. This is a result for one individual and different results may exist. The application of the Kobylarz-Bradley template, utilizing the forthcoming algorithms, yields a network of only one neuron that has the threshold function

F_XOR_ (**X**) = 1X_1_ + 1X_2_ - 2 X_1_ X_2_.

Being able to realize nonlinearly separable switching functions makes the Kobylarz-Bradley neuron model vastly more versatile than the linear neuron models currently deployed. Shown in Table 1 (Kobylarz and Kobylarz, 2023) are the amounts of possible logic functions for linearly separable functions L(n) (Gruzling, 2001) and the total number of logic functions T(n), with respect to the number (n) of axonal inputs. The quantity of linearly separable functions (L(n)) is cited in reference (Gruzling, 2001). The quantity of total logic functions (T(n)) is computed in reference (Kobylarz and Kobylarz, 2023). The final column of Table 1 is the ratio of these two numbers.

**Table 1:** Limitation of the AI linear neuron model (Kobylarz and Kobylarz, 2023).

***n***	**Linearly separable logic functions *L*(*n*) (Gruzling, 2001)**	**Total logic functions T(n)**	**Ratio T(n)/L(n)**
1	4	4	1
2	14	16	1.143
3	104	256	2.462
4	1,882	65,536	34.82
5	94,572	4,294,967,296	45,415
6	15,028,134	1.84467440737 × 10^19^	1.227 × 10^12^
7	8,378,070,864	3.40282366921 × 10^38^	4.062 × 10^28^

The exceedingly rapid growth of T(n), as shown in Table 1, illustrates the weakness of the currently deployed linear neuron model. For n = 7 there are greater than 4.06 d7 10^28^ (41 octillion) times more functions available than those that can be realized by an AI linear neuron model deployment. Even more startling is that a human neuron receives an average 10^3^ to 10^4^ inputs (Kriesel, 2007) from other neurons, suggesting the percentage of linearly separable functions is essentially zero when compared to a neurological neuron's capability.

**Figure 2:**
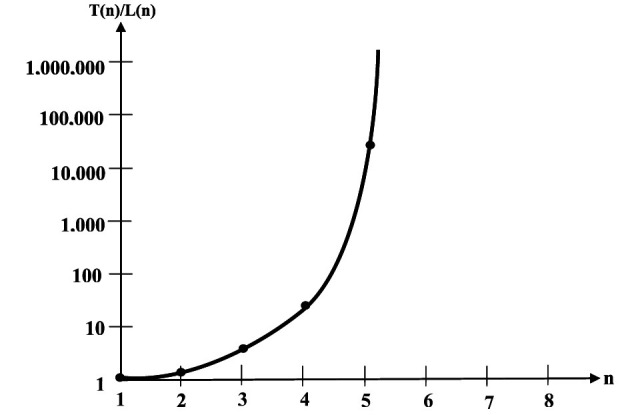
Growth of total function proportions (Kobylarz and Kobylarz, 2023).

A partial plot of the ratio vs. the number of inputs is shown in Figure 2 (Kobylarz and Kobylarz, 2023). Notice that the ordinate has a logarithmic scale. Logarithmic scales will plot exponential growth as a straight line. Even with the logarithmic scale the plotted rate of growth is much more rapid than exponential; suggesting that for thousands of inputs, this ratio is far beyond astronomical.

Adopting neuron models from the Kobylarz-Bradley template can be viewed as an increase of versatility over the strictly linear neuron model. This means that a template model of seven inputs is 4.06 × 10^28^ times more versatile than a linear model of seven inputs. Seven inputs represent the “tip of the iceberg,” since the AI neuron models have hundreds of inputs. The functional capability of AI neuron models is an infinitesimal portion of the possible functions.

## 2 Basic concepts to use the kobylarz-bradley template

**Figure 3:**
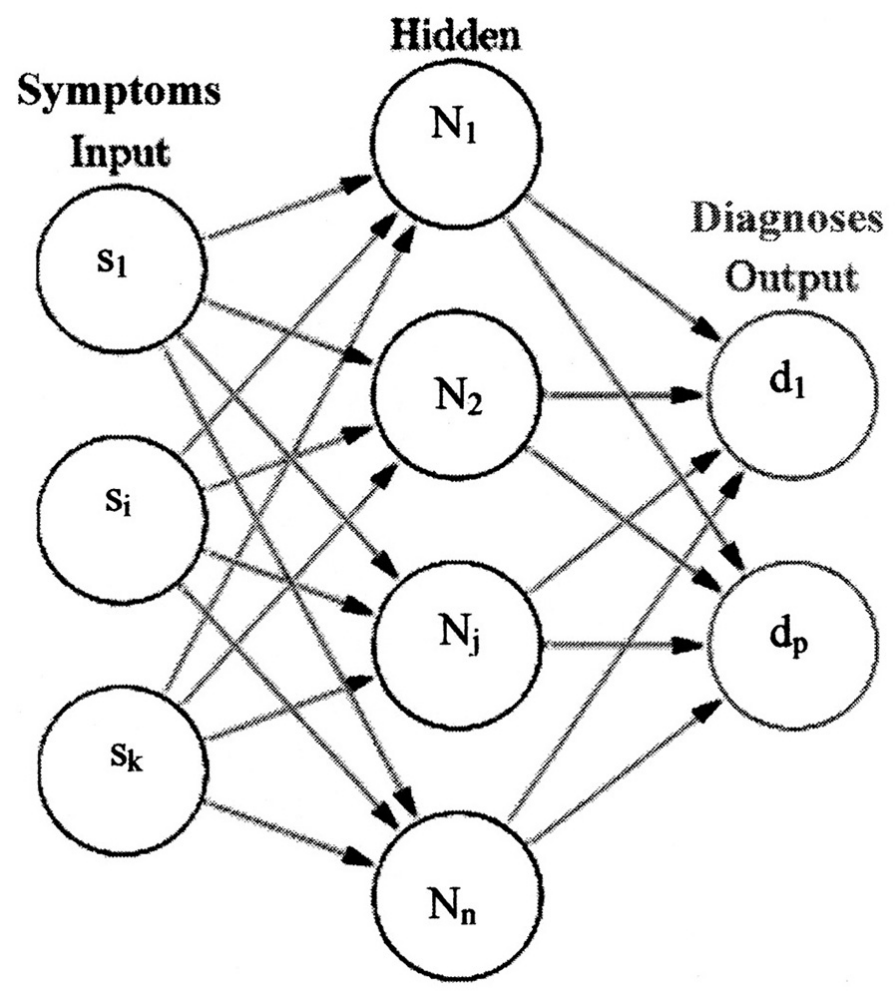
A simplified representation of a neural net.

The application of disease diagnosis from symptoms will be used to demonstrate how the Kobylarz-Bradley template is used. A simplified representation of a neural network, commensurate to this application, appears in Figure 3 (Neural Network, 2013). Some annotations are made to the original illustration for association with this application.

A set of symptoms, shown by the set “{s_1_, …, s_i_, …, s_k_}” (inputs), are presented to a trained neural network. The neurons operate on the symptoms to determine one or more diagnoses. The set of diagnoses are shown as “{d_1_, …, d_p_}” (outputs). Although only one hidden or deep layer is shown in Figure 3, many hidden layers are used in current AI systems. These networks are termed Deep Neural Networks (DNN). However, the newly presented concepts of this paper only use the input layer neurons. The outputs of these neurons represent diagnoses. Deep neural networks, using McCullough-Pitts neuron models, are considered to demonstrate performance improvements yielded by neuron models derived from the Kobylarz-Bradley template.

A label convention for variables is adopted within this paper. Lower case variables represent the binary digital axonal inputs/outputs of a neuron model. Upper case letters are used for the neuron model's internal operations. The letter “x” is used as the general neuron model's input. For the symptoms/diagnosis application, the letter “s” is used in lieu of “x” for the neural network's initial input. The letter “d” is used for the neural network's final output. The use of “s” and “d” are intended to emphasize this application of the neural network.

An “s_i_” is a label that associates a textual and/or auditory and/or visual representation of a symptom entered during a neural network's training. Likewise, “d_i_,” is a variable label that associates the choice of representation of a diagnosis entered during a neural network's training. After a representation is entered, the algorithm records this for future retrieval and ascribes the associated binary variable labels.

The general neuron model's input variable label, used within the algorithm, is “x_i_.” Hence, it is applicable to any application and to any layer. Because sets of one or more inputs exist in a neural network, the following representations are defined for {**s**} and {**x**}:


(6) $$\begin{array}{c} \left\{\text{s}\right\}=\left\{{\text{s}}_{\text{i}},\ldots ,{\text{s}}_{\text{k}}\right\} \end{array}$$

(7) $$\begin{array}{c} \left\{\text{x}\right\}=\left\{{\text{x}}_{\text{i}},\ldots ,{\text{x}}_{\text{k}}\right\} \end{array}$$

Similarly a neuron model's output variable representation, within the algorithm, is “u_i_.” The use of “u_i_” is intended to be general. Hence, related to any application and to any layer. To underscore the final layer's diagnoses outputs, “d_i_” is substituted for “u_i_.” But note that any portion of the algorithm, that has “u_i_,” is applicable to the final layer; i.e., “d_i_” is to be substituted. Because sets of one or more outputs exist in a neural network, the following representations are defined for {**d**} and {**u**}:


(8) $$\begin{array}{c} \left\{\text{d}\right\}=\left\{{\text{d}}_{\text{h}},\ldots ,{\text{d}}_{\text{p}}\right\} \end{array}$$

(9) $$\begin{array}{c} \left\{\text{u}\right\}=\left\{{\text{u}}_{\text{h}},\ldots ,{\text{u}}_{\text{p}}\right\} \end{array}$$

The algorithms, for applying the Kobylarz-Bradley template, use the letters “X” and “W” as the internal neuron model variables. Both will possess subscripts, as shown in the Equation 4. A transformation of signals occurs at synapses of a neuron. The transformation is represented by the following relationship:


(10) $$\begin{array}{c} {\text{X}}_{\text{j}}=\{\begin{array}{c} 0\Leftrightarrow {\text{x}}_{\text{j}}=0\\ 1\Leftrightarrow {\text{x}}_{\text{j}}=1 \end{array} \end{array}$$

Observe in Figure 3 that all inputs are shown connected to all first hidden layer neuron models and all final hidden layer neuron models are shown connected to all output neurons. Within hidden layers, all preceding hidden layer neuron models are connected to all succeeding hidden layer neuron models, until the output is reached. For us, the hidden layers are not “hidden,” but can be observed when a need exists. Furthermore, the connections are “available,” meaning that the algorithm places available connections into use, as needed. Otherwise, unused connections do not appear in an application resulting from training. This results in greatly simplified neural networks from using the algorithms to be described.

For current AI systems, properties of the deep layer neurons are unknown. In contrast, the presented methodology includes an address to identify a particular neuron of the network to retrieve a neuron's properties. This facility is important, especially during training. To do this, the network will be a rectangular array of neuron models to allow for the matrix notation of row “r” and column “c” pairs. Each column (“c”) identifies a hidden layer. By identifying the pertinent neuron model with the (r, c) pair values, it becomes possible to add/modify/remove a neuron's terms. Otherwise, the data remains permanently stored. The function “N(r, c)” is used as the address of a neuron model in the neural network. This permits the use of the template in Equation 4 to enact these changes to the addressed neuron model's threshold function terms. Details of “N(r, c)” and its accompanying neural network is the subject matter of a paper under preparation.

At its inception, all neurons of the network have no designated terms (the neuron models are empty) and no connections are in “use” (all are merely available). The terms are created during network training. An exception exists for input (sensory) neurons (column “0”). Sensory neurons are transducers that convert a stimulus to the binary axonal neurotransmission, recognized by internal neurons.

The internal neurons, according to Figure 3, are within the hidden layers. Therefore, an input neuron of layer 0 has its operation a' priori known. For example, the transducer (input neuron) converts the equivalent of a keyboard signal to a digital representation $“{\left({\text{S}}_{\text{i}}\right)}^{\prime \prime }$ that conforms to the internal neuron format.

It will be assumed that such a network has been built and the algorithms relates to the training of the neuron models in the network. Training encompasses the incorporation of neuron model operations derived from the Kobylarz-Bradley template. Hence, a reader's referral to the Kobylarz-Bradley template (Equation 1) is essential during the algorithms' description.

**Figure 4:**
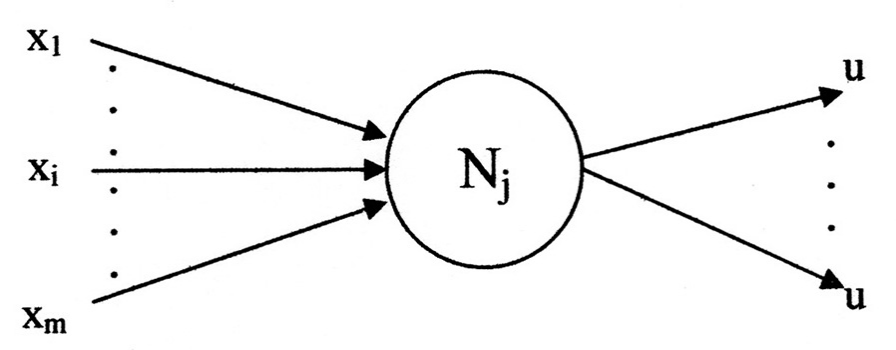
A graphical representation of a neuron model.

Figure 4 illustrates a general neuron model of the network. The inputs are represented by the set

“{X_1_, …, *X*_*i*_, …, *X*_*m*_}” and the single value output identified by “u.” The output may branch to multiple destinations. Each destination receives the same value of “u.” The variable “x” represents input generality with respect to the network layers. For this application the variable “s,” of Figure 3, is used as a specific case of “x.” The variable “s” identifies the initial inputs which are used by the first layer. Likewise, the general output symbol for all layers is “u.” In this application substitutes “d,” of Figure 3, for the final layer output.

A neuron model, in general, has two functional components (McCulloch and Pitts, 1943). The axonal input and output portions are associated with a switching function, shown as f_n_({**x**}). Its variables are binary (digital); i.e., variables possess one of the two per unit values “0” or “1.” The other functional component is an analog threshold function shown as F_n_({**X**}). The two functions interrelate to provide an axonal output, computed according to:


(11) $$\begin{array}{c} {\text{f}}_{\text{n}}\left(\left\{\text{X}\right\}\right)=\{\begin{array}{ccc} 1 & \Leftrightarrow & {\text{F}}_{\text{n}}\left(\left\{\text{X}\right\}\right)\geq \text{θ}\\ 0 & \Leftrightarrow & {\text{F}}_{\text{n}}\left(\left\{\text{X}\right\}\right)<\text{θ} \end{array} \end{array}$$

Where: f_n_ is the neuron's end to end logic function,

F_n_ is an analog threshold function performed by a neuron's cell,

**θ** is a threshold value contained within a neuron's cell,

{**x**} = {x_1_, …, x_m_} is the set of pre-synaptic values having digital components,

{**X**} = {X_1_, …, X_m_} is the set of post-synaptic values having analog components,

{**x**} = {**X**}, the two sets have equal per unit values,

⇔ represents “if and only if” hence, the inverse exists.

Equation 11 indicates the relation between f_n_({**x**}) and F_n_({**X**}). The distinction of the two functions is that f_n_({**x**}) represents the effective switching function between a dendritic input {**x**} and the axonal output f_n_({**x**}). However, the binary digital output computation is made internally to the neuron model by F_n_ ({**X**}), according to the inequalities of Equation 11. To make this computation, the binary digital input {**x**} is converted post-dendrite to the analog {**X**}. The conversion preserves the values of “0” and “1.”

An important property is that the threshold (**θ**) per unit values are always either “0” or “1” (Kobylarz and Kobylarz, 2023). A zero threshold value results in an unstable neuron and will not be considered in this paper. For stable systems, the threshold has a per unit value of “1.” This conforms to the experimental report that the neurological threshold variation is less than 100% (Yu et al., 2008); signifying that it cannot even double. This variation limit implies “and” functions would not be possible for neurological neurons if they could only perform linearly separable functions. The significance of this limitation illustrates that the currently deployed neuron models are a poor representation of a neurological neuron, as their thresholds need to at least equal “2” per unit to perform an “and” function. The neuron model proposed here is consistent with the neurological neuron's stable threshold of “1” per unit. For this reason, “**θ** = 1” is an initial condition of this methodology. Another initial condition is that all term weights are equal to “0”; which means no terms exist.

The template used to determine F_n_({**X**})is shown in Equation 4. Pertinent template terms are selected and term weights are assigned during training. At the inception of training, all weights have a zero (0) value; meaning none of the equation terms are yet used. Terms of the template will be selected by the assignments of non-zero weight values during the training phase. The other phase is the neural network deployment of neuron models that resulted from the training algorithm. Neuron model weights may also be changed, added, or removed during the deployment. The algorithm for deployment changes are variations of the training algorithms and will be described in an ensuing paper.

The medical diagnosis' training algorithm represents the iterative application of known ({s_1_, …, s_m_}, {d_1_, …, d_p_}) input/output set pairs to the neural network. While training, each time a new input set appears, an input neuron is selected, having the commensurate interconnections to the first hidden layer neurons that yield a path to an output d_i_ that belongs to “{d_1_, …, d_p_}.” Likewise, each time training indicates a new output, the output is established, having the commensurate interconnections to the final hidden layer.

## 3 Algorithms for the Kobylarz-Bradley template

Three algorithms are presented to establish a neuron model from the template. Execution of these algorithms represents training. The algorithm, named “Affirmation,” is used to select template terms for which the neuron output is to be “1.” The algorithm, named “Refutation,” is used to select template terms for which the neuron output is to be “0.” As will be seen, an execution of “Affirmation” can result in having other input sets also result in a “1” output. Should a “0” output be required for such an input set, the “Refutation” algorithm must be executed for the input set. The third algorithm “Subsumption” is used to remove superfluous terms in “F_n_({**X**}).”

It is important to note that at the beginning of training F_n_({**X**})contains no terms (the initial condition of all term weights equal to “0”). Hence, the output is “0” for all inputs (recall the initial condition of “**θ** = 1”). Therefore, “Refutation” is only necessary, if “Affirmation” has caused an input set to erroneously have a “1” output. Clarification will be made later.

A third possibility for an input set is a “don't care ($\underset{,}{\overset{,}{\omega }}$)” output. Because the output doesn't matter, no training is necessary for these input sets.

The algorithms are repeated for each successive output (u_*i*_) of an input/output set pair

({x_1_, …, x_m_}, {u_1_, …, u_*p*_}) presented during training. The variables are represented by {**x**}, {**X**}, and {**u**} for generality, as the three algorithms apply to all layers. To relate the diagnosis application's examples, the first layer variable labels **{s}**, **{S}** and **{d}**, for the final layer, are substituted for the general variables {**x**}, {**X**}, and {**u**}.

The first algorithm considered is named “Affirmation.” An execution of Affirmation provides a term to the neuron model's threshold function F_n_({**X**}), when the term is required for the axonal output. The input to Affirmation includes the input set “{x_i_, …, x_m_}” for which it is desired to have:


(12) $$\begin{array}{c} {\text{f}}_{\text{n}}\left({\text{x}}_{\text{i}},\ldots ,{\text{x}}_{\text{m}}\right)=1 \end{array}$$

The current threshold function F_n_({**X**}) represents the other input to Affirmation. If a change is made, F_n_({**X**}) is returned to the invoker by Affirmation. Otherwise, no change is made. The symbol “⊕” represents the “exclusive or” operation.

If the neuron is empty (first time Affirmation is used), the temporary assignment of F_a_({**X**}) in step 1 will also be empty until a term has been assigned. Observe that Affirmation assigns a value to W_a{X}_ only when there is an incorrect evaluation of “F_a_({**X**}).” The (No-op) is a flag sent to the invoker that means no changes were made to F_n_({**X**}).

**NEURON MODEL ALGORITHM 1/3 - AFFIRMATION**

To have f_n_ (x_i_, …, x_m_) = 1, Affirmation assigns a value to W_a{**X**}_ for the term

W_a{**X**}_ X_i_ … X_m_ according to the following procedure:

1) (Assign (F_a_ ({**X**}) = F_n_ ({**X**})) = > f_a_ ({**x**}) = f_n_ ({**x**})),

2) Apply the input {x_1_, …, x_m_ to f_a_ ({**x**}),

3) ⊕ 4)

3) (F_a_ ({**X**}) < **θ**) **= >** (Assign the smallest positive integer to

W_a{**X**}_∍: F_a_ ({**X**}) + W_a{**X**}_ **≥** **θ**),

**= >** (Assign F_n_ ({**X**}) = F_a_ ({**X**}) + W_a{**X**}_ X_i_ … X_m_)

4) (F_a_ ({**X**}) **≥** **θ**) = > (No-op)

It is possible that “F_a_({**X**}) ≥ **θ**” prior to the execution of step 3. Step 3 will not be executed and step 4 will be executed instead. This results in not having an additional term entered into F_a_({**X**}). The inequality of “F_a_({**X**}) ≥ **θ**” happens when a set “{x_i_, …, x_m_}” was previously processed by Affirmation and the set “{x_i_, …, x_m_, x_j_, …, x_k_}” is afterwards processed by Affirmation. Having the term “W_a{__**X**__}_ X_i_ … X_m_” already within “F_a_({**X**})” results in “f_n_ (x_*i*_, …, x_m_, x_j_, …, x_k__)_ = 1” or

“F_a_({**X**}) **≥** **θ**.” That is, the following relationship precludes adding a new term:


(13) $$\begin{array}{c} \left\{{\text{x}}_{\text{i}},\ldots ,{\text{x}}_{\text{m}},{\text{x}}_{\text{j}},\ldots ,{\text{x}}_{\text{k}}\right\}⊃\left\{{\text{x}}_{\text{i}},\ldots ,{\text{x}}_{\text{m}}\right\} \end{array}$$

That is, the execution of Affirmation to have f ({x_i_, …, x_m_}) = 1 will cause

f ({x_i_, …, x_m_, x_j_, …, x_*k*__}_) = 1. However, it may be desired to have f ({x_i_, …, x_m_, x_j_, …, x_k__}_) = 0. The remedy of such an error is the Refutation algorithm to provide a threshold term for the input set

“{x_i_, …, x_m_, x_j_, …, x_k_}.”

The template term for the Refutation is “W_r{__**X**__}_X_i_ … X_m_ X_j_… X_k_.” The “Refutation” algorithm has the role of assigning a value to “W_r{__***X***__}_” to make:


(14) $$\begin{array}{c} {\text{F}}_{\text{n}}\left(\left\{\text{X}\right\}\right)+{\text{W}}_{\text{r}\left\{\text{X}\right\}}{\text{X}}_{\text{i}}\ldots {\text{X}}_{\text{m}}{\text{X}}_{\text{j}}\ldots .{\text{X}}_{\text{k}}.<\theta \end{array}$$

**NEURON MODEL ALGORITHM 2/3 - REFUTATION**

The Refutation input is the set {x_h_, …, x_p_} and the current neuron model

function F_n_ ({**X**}).

Refutation assigns a value to W_r{**X**}_ for the term W_r{**X**}_ X_h_ … X_p_ according

to the following procedure:

1) Assign (F_r_ ({**X**}) = F_n_ ({**X**})) ⇒ (f_r_ ({**x**}) = f_n_ ({**x**})),

2) Apply the input {x_h_, …, x_p_} to f_r_ ({**x**}),

3) ⊕ 4)

3) (f_r_ ({**x**}) = 1) = > (Assign the smallest magnitude negative integer

to W_r{**X**}_∍: F_r_ ({X}) + W_r{X}_ < **θ**),

= > (Assign F_n_ ({**X**}) = F_r_ ({**X**}) + W_r{**X**}_ X_h_ … X_p_)

4) (f_r_ ({**x**}) = 0) = > (No-op)

During the refutation procedure, the notation “F_r_({**X**})” is assigned the neuron's threshold function, prior to the inclusion of the refutation term. If the Refutation conditions are satisfied, “F_n_({**X**})” will include the Refutation term within its threshold function. Also, for a Refutation term to be evaluated properly, the Affirmation term(s) that are refuted must first be determined.

The general Refutation input set is shown as {x_h_, …, x_p_} and the current neuron model threshold function is F_n_({**X**}). Prior to the addition of a refutation term, when one or more Affirmation input sets are included within {x_h_, …, x_p_}, then f_n_(x_h_, …, x_p_) = 1. This will provide a convenient means to determine inclusion of Affirmation terms and the evaluation of the weight of the Refutation term.

Step 3 is not executed when “f_r_ ({**x**}) = 0” (f_n_ ({**x**}) = 0) and step 4 is executed instead. Having

“f_r_ ({**x**}) = 0” implies that either Refutation is not necessary, due to no inclusion, or term had previously been incorporated within F_n_({**X**}) for the prescribed input.

Execution of Refutation may cause an erroneous result when the sequence with Affirmation is improperly executed. Consider that a pair of terms has been refuted and one of the terms is affirmed. If the affirmed term is also paired with a third term for which the latter pair is affirmed, the weight applied by Affirmation may be erroneous. An example of this error and a mitigating procedure will be provided later.

An optional algorithm deals with the removal of superfluous terms. A set of affirmed symptoms of the form “{x_i_, …, x_m_, x_j_, …, x_k_}” may be considered superfluous when the set “{x_1_, …, x_m_}” was also affirmed for the same inputs. Superfluous terms appear when the term (from the input “{x_i_ … x_m_ x_j_ … x_k_}”) is processed by Affirmation prior to an included term (from the input “{x_i_, …, x_m_}”). When the input “{x_i_ … x_m_ x_j_ … x_k_}” is applied, it will cause the term “W_a{__**X**__}_ X_i_ … X_m_” within the “F_n_({**X**})” to also be evaluated. When the term “W_a{__***X***__}_ X_*i*_ … X_m_” suffices for the result “F_n_({**X**}) ≥ **θ**”, then “W_s{__**X**__}_ X_i_ … X_k_ X_j_ … X_k_” may be considered superfluous and removed by assigning “W_s{__**X**__}_ = 0.” The algorithm Subsumption is used to make such an assignment and remove superfluous terms in “F_n_({**X**}).” If the option is selected, Affirmation will invoke Subsumption following the assignment of a new term “W_a{X}_ X_i_ … X_m_” to “F_n_({**X**}).” Subsumption receives “W_a{__**X**__}_ X_i_ … X_m_” and “F_n_({**X**})” as its inputs.

**NEURON MODEL ALGORITHM 3/3 - SUBSUMPTION**

After Affirmation assigns W_a{X}_ X_i_ … X_m_ to F_n_ ({**X**}) the steps

for Subsumption follow:

1) Assign F_s_ ({**X**}) = F_n_ ({**X**}) - W_a{X}_ X_i_ … X_m_,

2) Repeat for each term (W_s{X}_ X_j_ … X_k)_ of F_s_ ({**X**}),

3) ⊕ 4)

3) {X_j_, …, X_k_} ⊃ {X_i_, …, X_m_} = >(W_s{X}_ = 0 for F_n_({**X**}] term W_s{X}_X_i_…X_k_)

(Removes term),

4) ({X_j_, …, X_k_} ⊅ {X_i_, …, X_m_}) = > (No-op),

Keeping both “W_a{__**X**__}_ X_i_ … X_m_” and “W_s{__**X**__}_X_i_ … X_m_ X_j_ … X_k_” in F_n_({**X**}) does not cause computation errors. The adverse consequences from superfluous terms include additional memory and processing during the program's application. However, searching for superfluous terms during training may be an exorbitant effort, outweighing the application's processing burden.

An example deploying these three algorithms will now be provided by the use of tables. Because symptoms are the example inputs, the symbols “{**S**}” and “{**s**}” will be used. Consecutive rows of a table represent the sequence of symptom “**{s}**” inputs, which are identified in the middle column. The column to the left presents the threshold function “**F**_n_(**{S}**)” prior to applying an input “{**s**}.” The column to the right presents the threshold function “**F**_n_(**{S}**)” after applying an input “{**s**}.” Table 2 has two sections corresponding to the first two algorithms. It represents the beginning of training. The training input sequence begins with Affirmation. Having Affirmation precede Refutation is important because the affirmed set needs determination to be refuted.

**Table 2:** An example of the algorithms 1 and 2 executions.

	**Affirmation (1/3)**		
**Step**	**F**_n_ **({S}) before affirmation**	**Symptoms input**	**F**_n_ **({S}) after affirmation**
1	0 (No terms)	{s_1_}	S_1_
2	S_1_	{s_2_, s_3_}	S_1_ + S_2_ S_3_
3	S_1_ + S_2_ S_3_	{s_3_, s_4_, s_5_}	S_1_ + S_2_ S_3_ + S_3_ S_4_ S_5_
	**Refutation (2/3)**		
**Step**	**F**_n_ **({S}) before refutation**	**Symptoms input**	**F**_n_ **({S}) After refutation**
4	S_1_ + S_2_ S_3_ + S_3_ S_4_ S_5_	{s_1_, s_2_, s_3_}	S_1_ + S_2_ S_3_ + S_3_ S_4_ S_5_ – 2 S_1_ S_2_ S_3_

Table 3 illustrates a sequence of two Affirmations for which the first Affirmation is superfluous and Subsumption is applied to remove the superfluity. Table 3 is shown as an extension to Table 2.

**Table 3:** An example of subsumption.

**Affirmation (1/3)**			
**Step**	**F**_n_ **({S}) before affirmation**	**Symptoms input**	**F**_n_ **({S}) after affirmation**
5	S_1_ + S_2_ S_3_ + S_3_ S_4_ S_5_ – 2 S_1_ S_2_ S_3_	{s_3_, s_4_, s_6_, s_7_, s_8_}	S_1_ + S_2_ S_3_ + S_3_ S_4_ S_5_ – 2 S_1_ S_2_ S_3_ + S_3_ S_4_ S_6_ S_7_ S_8_
6	S_1_ + S_2_ S_3_ + S_3_ S_4_ S_5_ – 2 S_1_ S_2_ S_3_ + S_3_ S_4_ S_6_ S_7_ S_8_	{s_4_, s_8_}	S_1_ + S_2_ S_3_ + S_3_ S_4_ S_5_ – 2 S_1_ S_2_ S_3_ + S_3_ S_4_ S_6_ S_7_ S_8_ + S_4_ S_8_
	**Subsumption (3/3)**		
7	{s_3_, s_4_, s_6_, s_7_, s_8_} ⊃ {s_4_, s_8_}		
	W_s_ = 0 for W_s_S_3_ S_4_ S_6_ S_7_ S_8_		
	F_n_ ({**S**}) = S_1_ + S_2_ S_3_ + S_3_ S_4_ S_5_ – 2 S_1_ S_2_ S_3_ + S_4_ S_8_		

A digression will now be made to demonstrate the improved efficiency of using the Kobylarz-Bradley template. Consider the Table 3 product terms in F_n_({**S**}); e.g., “S_3_ S_4_S_5_.” These correspond to “and” gates. Also observe that in this training process, the “and” gate terms were determined by a single step. Training of McCulloch-Pitts neuron models (McCulloch and Pitts, 1943) will require at least three steps because a threshold of 3 is required and the increment per training step is “one.” Furthermore, “and” gates may have hundreds of inputs in an AI application, requiring over hundreds of iterations to acquire the required threshold value for its “and” gates. By comparison, the Kobylarz-Bradley template will still create the “and” gate term in one step for any number of “and” gate inputs.

**Figure 5:**
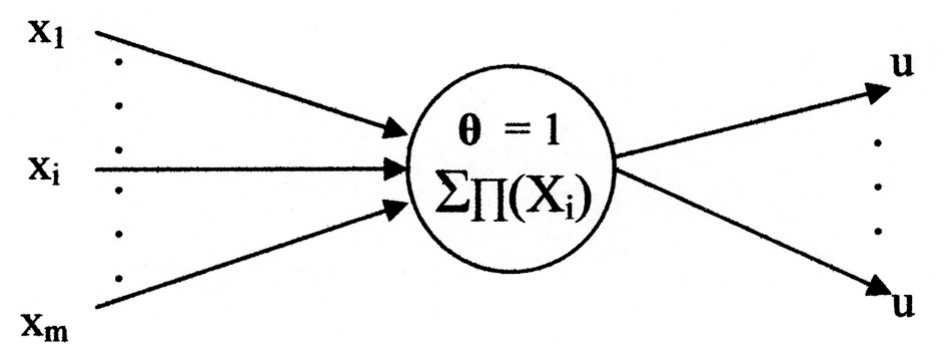
Kobylarz-Bradley neuron model graphical representation.

**Figure 6:**
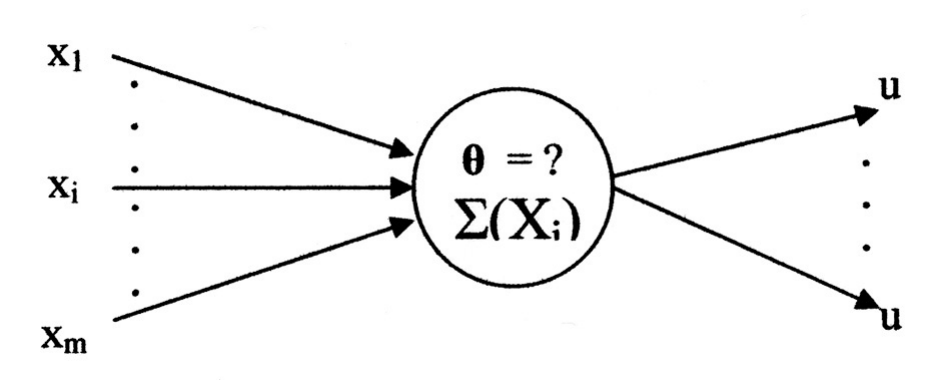
McCulloch-Pitts neuron model graphical representation.

Graphical representations to achieve the example's functionality for both Kobylarz-Bradley and McCulloch-Pitts neuron models will now be shown. The two neuron models will have the general representations shown in Figures 5, 6.

**Figure 7:**
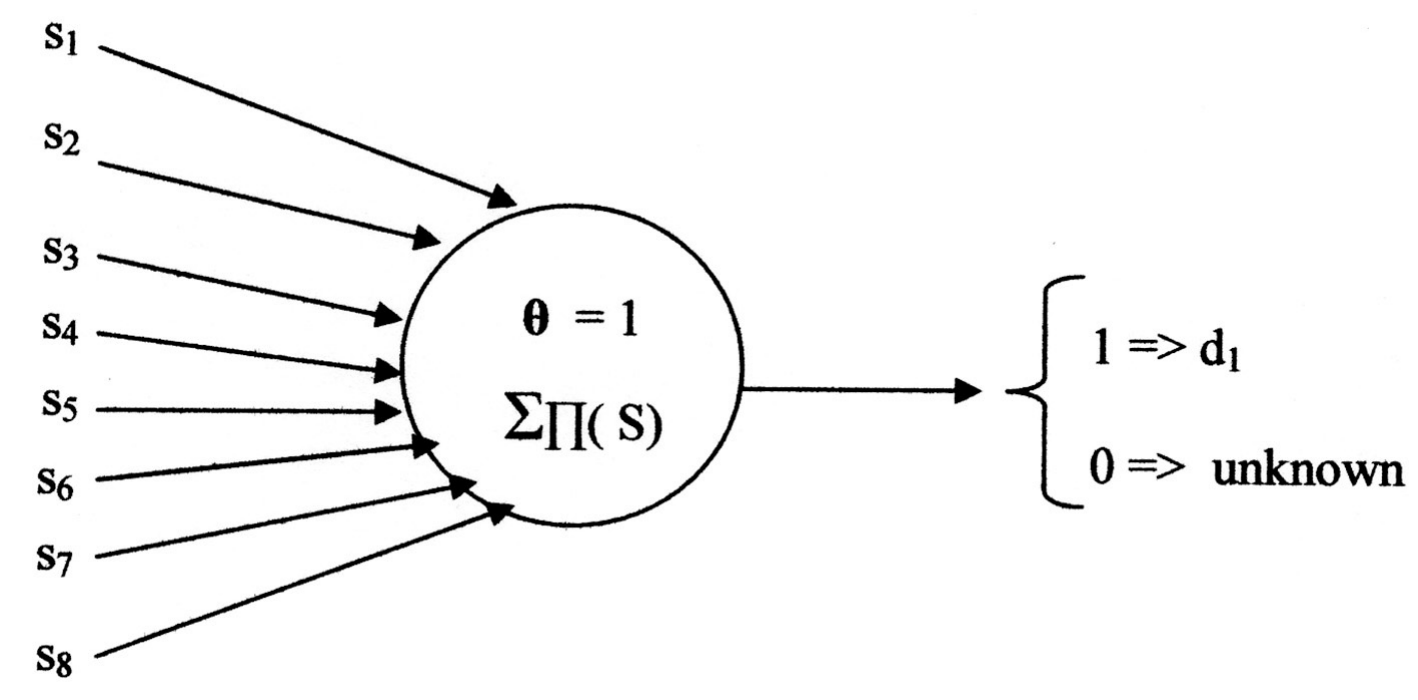
Neural network example using Kobylarz-Bradley neuron model.

Figure 7 illustrates the neural net using the Kobylarz-Bradley neuron model. Only one neuron is needed, having the threshold function:


(15) $$\begin{array}{c} {\text{F}}_{\text{n}}\left(\left\{\text{S}\right\}\right)={\text{S}}_{1}+{\text{S}}_{2}{\text{S}}_{3}+{\text{S}}_{3}{\text{S}}_{4}{\text{S}}_{5}-{2\text{S}}_{1}{\text{S}}_{2}{\text{S}}_{3}+{\text{S}}_{4}{\text{S}}_{8} \end{array}$$

**Theorem**

Application of the Kobylarz-Bradley template permits a network of a single neuron for each binary, digital network output.

**Proof**

In reference (Kobylarz and Kobylarz, 2023) it is proven that the Kobylarz-Bradley template represents any switching function of “n” variables. Also, the relationship of an input/output pair ({**s**}, d) is representable by a switching function. By applying the Kobylarz-Bradley template, the threshold function of a single neuron model is provided.

**Q.E.D**.

The cost of the Kobylarz-Bradley Neural network is one neuron model, one layer, one row, eight inputs, four products, four summations, and one threshold comparison. Also, it was generated by single iterations of three algorithms, having a total of seven steps. Most importantly, it provides the correct diagnosis for all symptom sets applied in training and will not provide a diagnosis for symptom sets that are not recognized. The contrast with existing neural nets, like ChatGPT (Lock, 2022), normally provide an answer, regardless of whether the answer is wrong or right. An erroneous diagnosis may be misconstrued as plausible and, if accepted, can result in an injury or even a fatality.

It is no surprise that one neuron suffices for the implementation. The Kobylarz-Bradley template provides the means to create a neuron model capable of performing any switching function. Hence, it can always yield a single neuron network. A description of creating networks of multiple neurons is later presented.

**Tautology**

A neural net's end-to-end functionality (input-to-output) is expressible by one or more switching functions.^1^

**Rational**

A neural net has binary digital inputs and a set of one or more binary digital outputs. Hence regardless of its internal complexity, the net's end-to-end functionality is representable by a switching function for each output. Furthermore, it is essentially a propositional calculus axiom that every logic function (switching function) has a corresponding truth table (table of combinations) and conversely. Each specific neural net's input set, in combination with the value of an associated output, represent a truth table row. The corresponding truth table is formed by having its rows correspond to all possible combinations of true/false values, accompanied by an output value. Many methods exist by which a logic function can be inferred from such a truth table.

With respect to the algorithm, Affirmation creates rows for which an output is “1.” Refutation creates rows having a “0” output value. By the definition of the template, all remaining rows will have a “0” output value. A switching function representation that corresponds to the example is:


(16) $$\begin{array}{c} {\text{f}}_{\text{n}}\left(\left\{\text{s}\right\}\right)=\\ \;({\text{s}}_{1}+{\text{s}}_{2}{\text{s}}_{3}){\left({\text{s}}_{1}{\text{s}}_{2}{\text{s}}_{3}\right)}^{\prime }+\left({\text{s}}_{3}{\text{s}}_{4}{\text{s}}_{5}\right)+\left({\text{s}}_{3}{\text{s}}_{4}{\text{s}}_{6}{\text{s}}_{7}{\text{s}}_{8}\right)+\left({\text{s}}_{4}{\text{s}}_{8}\right) \end{array}$$

**Figure 8:**
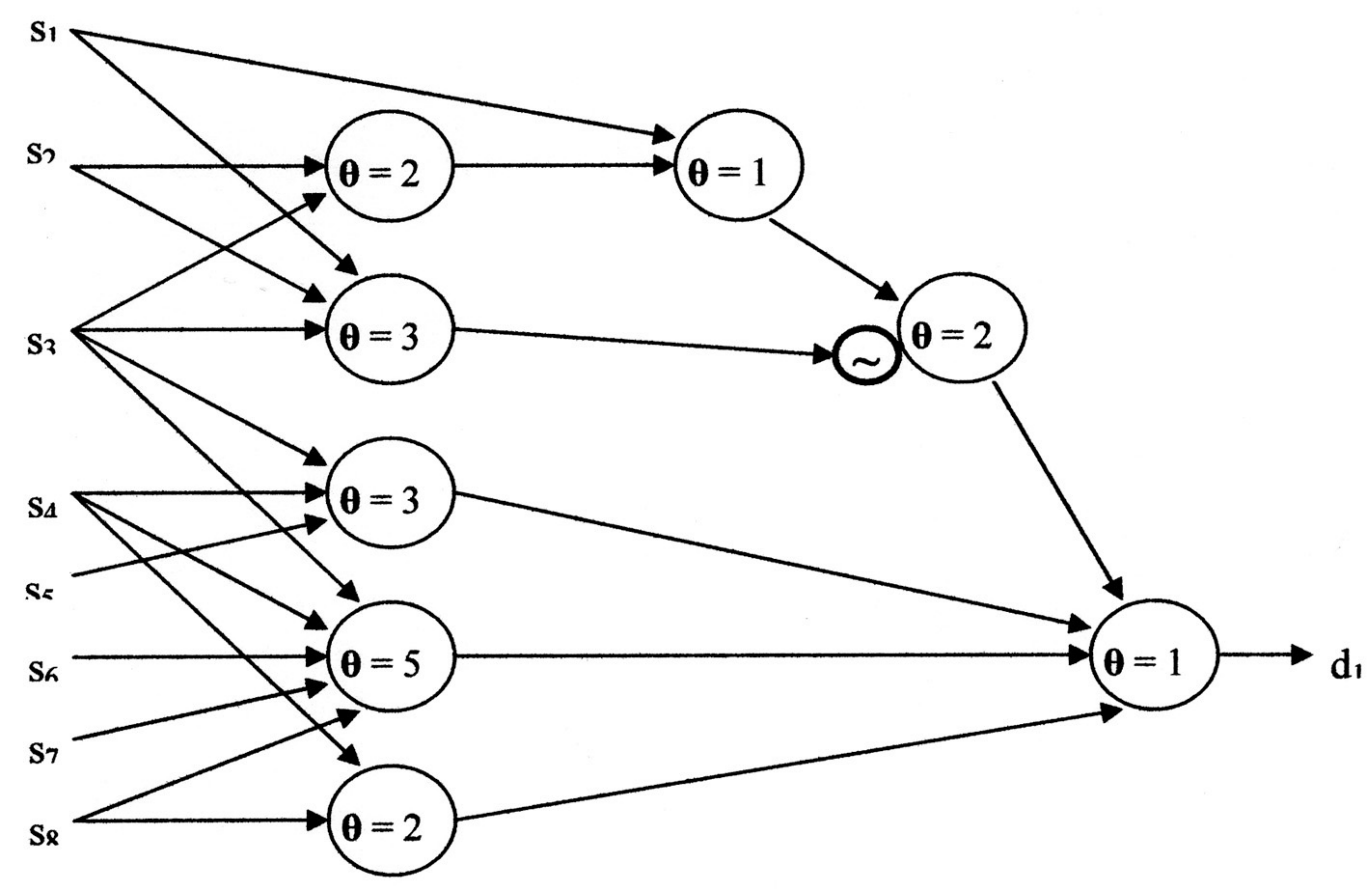
The neural network resulting from using the McCulloch-Pitts neuron model.

A neural network for Equation 16, using the McCulloch-Pitts neuron model, is shown in Figure 8. When **θ** = 1, the McCulloch-Pitts neuron model performs a logical “Or” function. The logical “And” function is performed by the McCulloch-Pitts neuron model when **θ** **≥** 2. The circle enclosing “**~**” represents an inhibitory synapse (logical negation).

Although simpler neural networks may exist, it is likely close to the complexity of Figure 8. The cost of the McCulloch-Pitts neural network is eight neuron models, four layers, 23 inputs, eight summations, one negation, and eight threshold comparisons. The versatility ratio of the Kobylarz-Bradley neuron model to the McCulloch-Pitts neuron model grows more rapidly than exponentially. For applications, inputs may number in the hundreds. Hence, neural network complexity, using the McCulloch-Pitts neuron model, will be horrendous compared to using the Kobylarz-Bradley model.

**Figure 9:**
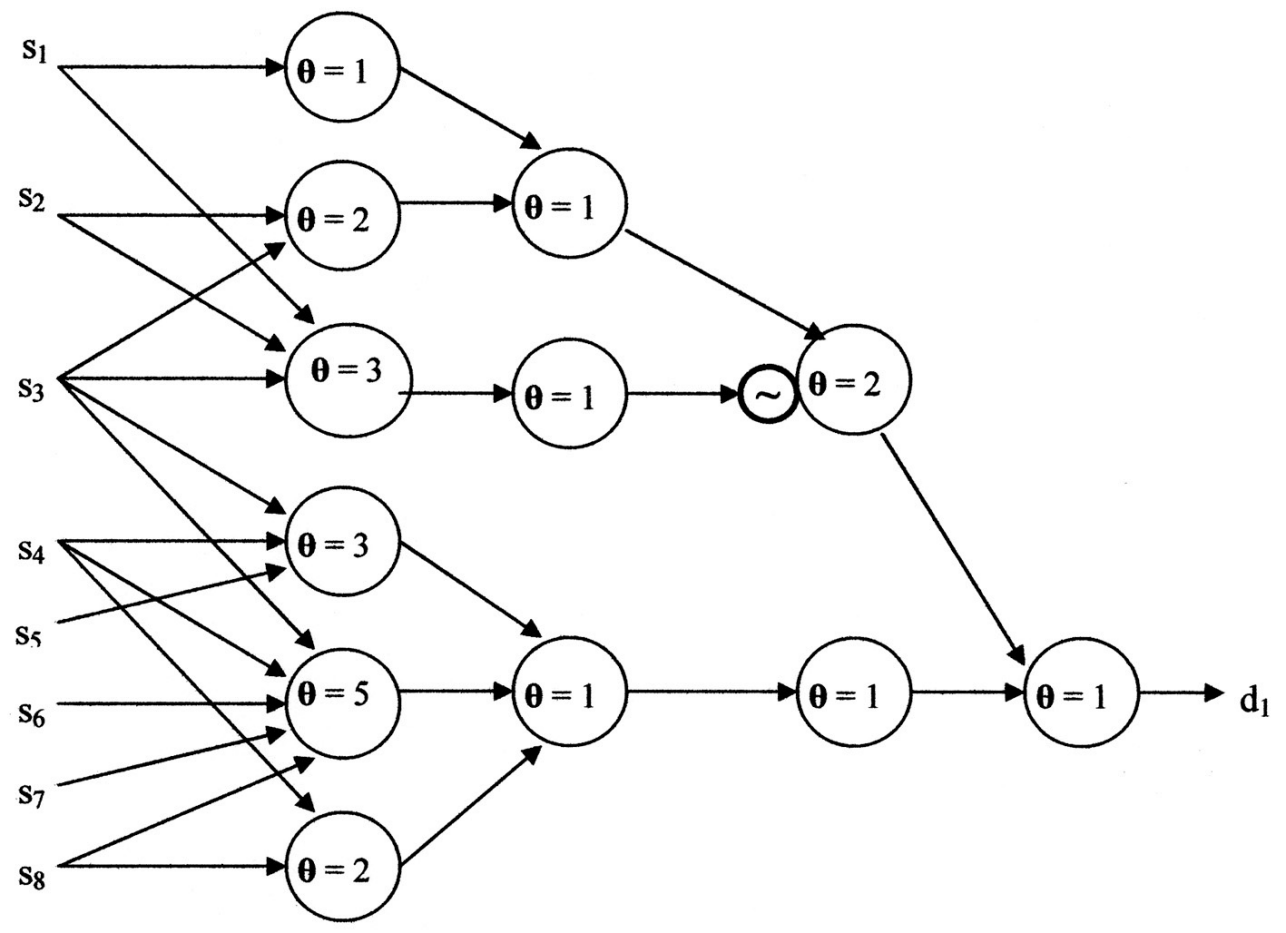
The neural network without skipping layers, using the McCulloch-Pitts neuron model.

The current neural networks' connectivity is established before training is begun. Hence, it is not possible to skip layers, as is shown in Figure 8. Figure 9 is a realization of the example when no layers can be skipped. When comparing Figure 9 to only one neuron, the value of the Kobylarz-Bradley model is obvious.

The switching function shown in Equation 16, demonstrates that algorithms, using the Kobylarz-Bradley neuron model, significantly decrease training intensity. No algorithm exists to directly create the McCulloch-Pitts neural network of Figures 8, 9. It needs to be established by “trial and error.” Although the trial and error process includes positive and negative reinforcement, having to establish the connectivity of the pertinent neuron models' web, including their thresholds and negations, represents a formidable undertaking. This will require much iteration, most likely in the thousands and yet not replicate the function perfectly. Furthermore, function errors or an omission, as the omission of (s4 s8) in Equation 16 and not in Equation 17, could result at the after training:


(17) $$\begin{array}{c} {\text{f}}_{\text{n}}\left(\left\{\text{s}\right\}\right)=\left({\text{s}}_{1}+{\text{s}}_{2}{\text{s}}_{3}\right){\left({\text{s}}_{1}{\text{s}}_{2}{\text{s}}_{3}\right)}^{\prime }+\left({\text{s}}_{3}{\text{s}}_{4}{\text{s}}_{5}\right)+\left({\text{s}}_{3}{\text{s}}_{4}{\text{s}}_{6}{\text{s}}_{7}{\text{s}}_{8}\right) \end{array}$$

The omission of the term “(s_4_ s_8_)” means that many possible symptom inputs will not determine the diagnosis. That is, the inadequate neural network manufactures an error by not possessing the correct switching function. Comparatively, the algorithm utilizing the Kobylarz-Bradley template will not manufacture additional errors. Any neural network error is due to the intrinsic error rate of the training symptoms/diagnosis set. If this set is perfect, the neural network error will be “0%.”

Indicated earlier was that the improper ordering of an Affirmation and Refutation may result in errors. The following example function illustrates how an error may occur:


(18) $$\begin{array}{c} {\text{f}}_{\text{n}}\left(\left\{\text{s}\right\}\right)=\left({\text{s}}_{1}+{\text{s}}_{2}+{\text{s}}_{3}\right){\left({\text{s}}_{1}{\text{s}}_{2}\right)}^{\prime }{\left({\text{s}}_{1}{\text{s}}_{3}\right)}^{\prime }{\left({\text{s}}_{2}{\text{s}}_{3}\right)}^{\prime }+{\text{s}}_{1}{\text{s}}_{2}{\text{s}}_{3} \end{array}$$

Assume that Affirmations are first performed on the term “**(**s_1_ + s_2_ + s_3_).” This will result in the following partial threshold function results:


(19) $$\begin{array}{c} {\text{F}}_{\text{a}}\left(\left\{\text{S}\right\}\right)={\text{S}}_{1}+{\text{S}}_{2}+{\text{S}}_{3} \end{array}$$

If an Affirmation is now performed on the term “s_1_ s_2_ s_3_,” no threshold function term will be added because of inclusion by the switching terms “{s_1_},” “{s_2_},” and “{s_3_}.”

When Refutations are afterwards performed for (s_1_ s_2_)′ (s_1_ s_3__)_′ (s_2_ s_3__)_′, the threshold function becomes:


(20) $$\begin{array}{c} {\text{F}}_{\text{r}}\left(\left\{\text{S}\right\}\right)={\text{S}}_{1}+{\text{S}}_{2}+{\text{S}}_{3}-{2\text{S}}_{1}{\text{S}}_{2}-{2\text{S}}_{1}{\text{S}}_{3}-{2\text{S}}_{2}{\text{S}}_{3} \end{array}$$

According to Equation 18, when s_1_ = s_2_ = s_3_ = 1, then f_n_({**s**}) = 1. However, Equation 20 yields F_r_({**S**}) = −3 < **θ**. This erroneous result is a consequence of the training sequence used. Rather than establishing an algorithm to properly sequence training steps, a simpler remedy is to run a test of all inputs following training. When an input fails to yield a “1” output, the Affirmation algorithm is performed for the failed input. Likewise, if an input fails to yield a “0” output, the Refutation algorithm is again performed for the failed input. The example's failure implies an Affirmation is needed for the input (s_1_ s_2_ s_3__)_. Performing Affirmation, for this input, yields the correct threshold function:


(21) $$\begin{array}{c} {\text{F}}_{\text{n}}\left(\left\{\text{S}\right\}\right)={\text{S}}_{1}+{\text{S}}_{2}+{\text{S}}_{3}-{2\text{S}}_{1}{\text{S}}_{2}-{2\text{S}}_{1}{\text{S}}_{3}-{2\text{S}}_{2}{\text{S}}_{3}+{4\text{S}}_{1}{\text{S}}_{2}{\text{S}}_{3} \end{array}$$

Due to sequence errors, such as for the preceding example, the following recommendation is made. Before the neural net is released as an application, testing of all inputs used during training is performed. Should any errors result, either Affirmation and/or Refutation will be conducted for the failed inputs, depending on the type of error.

The algorithms presented here create a neural network that, during implementation, yields the information for which it has been trained. That is, if an input set “{**s**}” matches the set of a training set diagnosis pair ({**s**}, d), the implementation will faithfully yield the diagnosis “d” of the pair. This means that the implementation accuracy is controlled by the adequacy and accuracy of the training diagnosis pairs.

If an input set “{**s**}” does not appear in any training set diagnosis pair, the implementation will yield an equivalent of “I don't know” or not provide a response, depending upon an application's initial conditions. It is possible to misconstrue the “I don't know” or no response as a negative diagnosis (possible false negative). Also, training's diagnosis pair ({**s**}, d) may inherently contain a false negative possibility or be erroneous. However, the algorithms will not manufacture false negative responses; so that the possibility of a false negative is the same as that contained by the training data. The currently used AI methodology may manufacture false negative outcomes.

Similarly, false positive diagnoses may result when the data inherently has a false positive possibility. The algorithms do not manufacture false positive outcomes. Again, the currently used AI methodology may manufacture false positive outcomes (hallucinations).

An implementation error rate wholly depends upon the training. Hence, training data needs to be as correct and comprehensive, as possible. No means are known to compute an exact error rate. Empirical testing represents a process for estimating an application's error rate. Prior to making an application publically available, thousands of known cases need to be run. The errors are to be observed and corrected by either Affitmation or Refutation. If the error rate was unacceptable, the process is repeated until a satisfactory error rate exists. After the final iteration, one can expect that empirical evidence suggests an acceptable accuracy. It is believed that the 92% average accuracy of current neural networks will be easily surpassed by Kobylarz-Bradley neural networks. Even a 100% empirical accuracy appears feasible for certain applications.

## 4 Preview of network generation

Heretofore, there has been no description of how a network of Kobylarz-Bradley neurons is generated. This work is now underway and will be the subject of a future paper. An early stage of this endeavor is the application of the neurological associative learning aspect of neuroplasticity (Carter, 2009). This neurological process will forge an axonal link between pairs of neurons that fire together following a common stimulus. Forming such links institutes network interconnections. Algorithms, emulating neurological associative learning, are planned.

**Figure 10:**
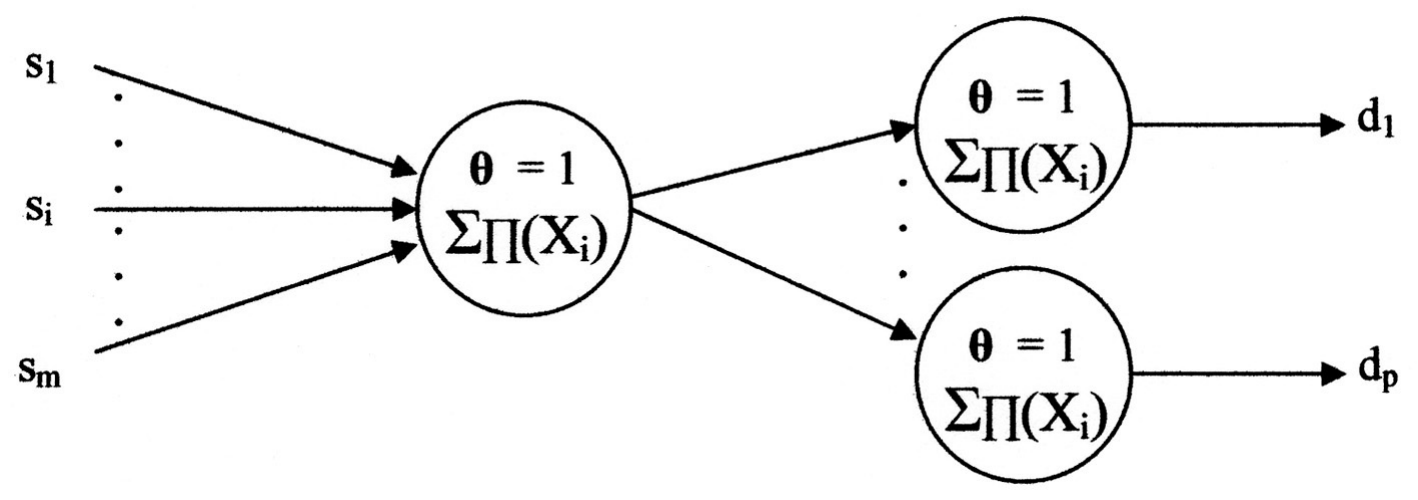
The primitive neural network implied by the algorithms.

The training input/output set pairs “({s_1_, …, s_m_}, {d_1_, …, d_p_})” imply a primitive neural network. As shown in Figure 10, only peripheral neuron models occur from execution of the algorithms.

**Figure 11:**
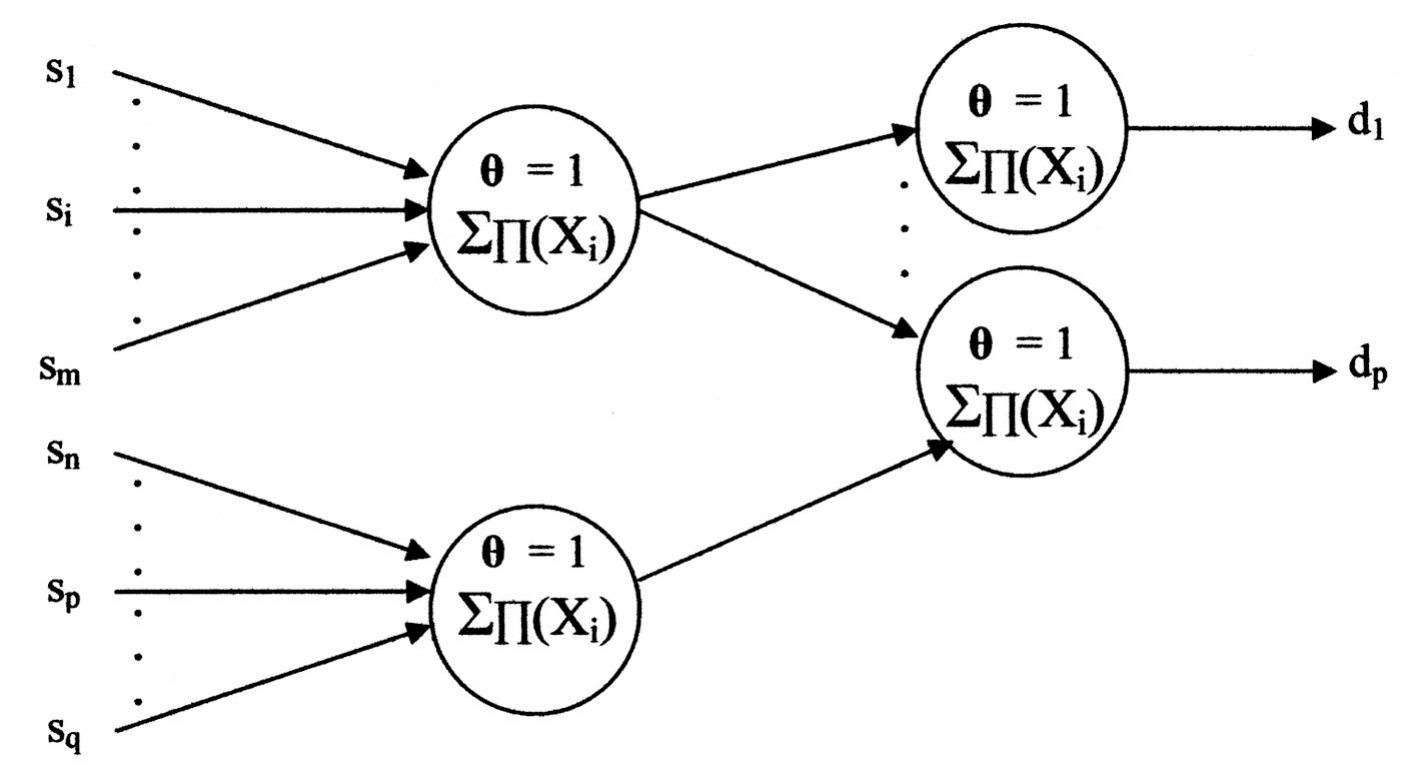
The primitive neural network sharing a diagnosis.

Should a different input set “**{S}**” have a common diagnosis, say “d_p_,” the network may appear as shown in Figure 11.

## 5 Discussion

### 5.1 Model's performance improvements

By only realizing linear separable switching functions, the McCulloch-Pitts neuron model has severely limited versatility. For seven variables, the ratio of available functions to linearly separable functions is 4.062 × 10^28^. As the number of variables increases, this ratio grows faster than exponentially. Being essentially infinitesimal for seven variables, McCulloch-Pitts neuron model applications are stymied from utility when tens or hundreds of variables exist. This results in enormous neural networks and many training days, with no hope of 100% accuracy.

When current neural networks are large, the parameter gradients become exetremely small and may vanish during training of deep neural networks. This slows down or stops learning in deep networks because weight updates are negligible. Rectified linear unit (ReLU) (Brownlee, 2020) provide a means to help mitigate this problem. Since this problem does not exist when using the Kobylarz-Bradley template, no mitigation procedure's are required.

It has been shown that the neurological neuron is capable of performing nonlinearly separable functions, which comes as no surprise. One expects that Mother Nature, with her infinite wisdom, would not mistakenly use the McCulloch-Pitts neuron model in her neural networks. Additionally, having a threshold value fixed at “1” per unit corresponds to neurological measurements made (Yu et al., 2008). Extrapolating, the neurological brain's efficiency is a persuasive indication that the incorporation of neurological modeling will lead to improved results. I attribute the better performance to a more neurologically accurate neuron model.

The neurological property of performing all switching functions is provided by the Kobylarz-Bradley template. This extensive neuron model versatility provides the means to establish much simpler networks. The example presented here shows the relative complexity of the McCulloch-Pitts neuron model network (Figure 9) compared to the Kobylarz-Bradley neuron model network of only one neuron.

Relative complexity represents one of two McCulloch-Pitts neuron model disadvantages. The other drawback is training, which is intended to establish neural networks that provide correct results. Figure 9 shows an example of such a network. However, current training algorithms do not create a network directly. They do so by trial and error. Having high accuracy from a trial and error process is very difficult to achieve. The hope is that the error rate will be low when the trained network is applied. The error rate for the McCulloch-Pitts neuron model networks averages 8% (Thompson et al., 2021). Although the AI implementation error rate varies with specific applications, it is used as a frame of reference here. Whereas, for an application's invocation of a trained input, the Kobylarz-Bradley neuron model network is anticipated to have much better than an 8% error rate.

It is speculated that thousands of training iterations may be necessary for a McCulloch-Pitts neural network to learn the example.

I propose a challenge to owners of AI systems, more advanced than one using a McCulloch-Pitts neural network, to train their AI system to perform one of this paper's examples. Reporting the performance results to me would be greatly appreciated.

### 5.2 Applications

I foresee many medical applications of the Kobylarz-Bradley neuron model networks. Work has embarked on recognition of atrial fibrillation. Because atrial fibrillation is a precursor of strokes, its early determination, and then proper treatment can preclude the devastating consequences of a stroke. The efficiency of the neuron models will result in small networks that consume little power. This suggests that electronic neural network chips, can be used by any device that observes the heart beat, to detect atrial fibrillation; e.g., a consumers' ordinary sphygmomanometer (blood pressure meter).

Another important application of the Kobylarz-Bradley neuron model networks is symptom recognition of epileptic seizures. The present practice of electroencephalography (EEG) recording contains many channels, requiring complicated and uncomfortable cranium connections. Studies are being conducted to determine the feasibility of using non-cerebral, time-series data to detect epileptic seizures (Hamlin et al., 2021). Patients wear sensors monitoring electrocardiography, electrodermal activity, electromyography, accelerometry, and audio signals. These physical manifestations accompany seizures and have the advantage of sensor use in a normal patient environment. The use of a Kobylarz-Bradley neuron model network can be trained to recognize combinations of sensor signals to detect seizures.

### 5.3 Further algorithm work

Study is now underway to build network layers beyond the input layer. They will not be hidden layers. Rather, neurological processes to create and connect neurons will be emulated. A related pursuit, which applies to establishing neuronal connections beyond the first layer, is to include an algorithm that provides a probability assessment of outputs, based on statistical data. Such an algorithm will not only yield statistical results for input/output data, but is also intended to indicate output probabilities for (input, output) pairs that have not appeared in training.

When networks of Kobylarz-Bradley neuron models exist, additional neurological properties can be introduced. An important neurological property is Hebbian Learning (Hebb, 1949). Hebbian Learning is a process in which synaptic connections between neurons are strengthened when they are simultaneously active. The stremgthened connections represent associative memory, which is a very pwerful lesrning means.

It has been observed that the spike (firing) rate of neurons can vary (Smith and Ratcliff, 2004). A possible reason for the variation is to invoke a state change. Human reactions to emergencies suggest that the brain is a state machine. That is, the present functionality is conditioned upon a concurrent state of cognition and a state change alters functionality. An apt phrase supporting this belief is “A state of shock.” When a person experiences a shocking experience, the reaction to a stimulus is usually quite different than a reaction when circumstances are normal and more predictable. This suggests that the functional relation to a stimulus is dependent upon an individual's cognitive state. An initial exploration has begun on introducing a state operation into a neural network's behavioral model.

Situations exist in which it is possible to learn while an application is being executed. An example of such situations and an algorithm to enact learning will be the subject of an ensuing paper.

Because funding to implement these findings has been twice denied, only theoretical research is being performed. I hope that there will be interest, by sources with funding, in pursuing the experimental development of a neural network having neuron models generated by the Kobylarz-Bradley neuron model template. The results of such an endeavor will either show that the algorithm has little value, or demonstrate that it is a major AI milestone, or something in-between.

## 6 Conclusion

It can be concluded that AI in medicine, including all specialties is still in the developmental phase, although some progress has been made, particularly in the recent past. For instance, prognosis as well as diagnosis (CRI Staff, 2025). This technology has great potential on a number of fronts, to include the diagnosis and treatment of patients, as well as in medical education and training. Given the serious consequences from any errors that might be made from inadequate machine learning models, guardrails must be put in place during its development. I feel that the Kobylarz-Bradley template will yield neural networks far more accurate and superior to those utilizing the McCulloch-Pitts model. Even so, results interpretation by appropriately trained physicians will continue to be necessary.

## Data Availability

The original contributions presented in the study are included in the article/supplementary material, further inquiries can be directed to the corresponding author.

## Footnotes

^1^This excludes having state variables within a neural network's operation. No publication has been found of an AI neural network in which state variables are used.
